# Supplementary material for: Deficiency of ValRS-m Causes Male Infertility in Drosophila melanogaster
Source: Int J Mol Sci. 2024 Jul 8;25(13):7489. doi: 10.3390/ijms25137489 (PMC11242588; doi:10.3390/ijms25137489)
Supplement: Supplementary file 1 [file ijms-25-07489-s001.zip › Tables S1 and S2.pdf]

Table S1. Summary of RNA sequencing data for six samples

Sample	Raw Reads	Raw Bases	Clean Reads	Clean Bases	Error Rate (%)	Q20 (%)	Q30 (%)
ValRS-m-RNAi -1	57999218	8757881918	57643064	8519866109	0.0249	98.11	94.15
ValRS-m-RNAi -2	53178026	8029881926	52863044	7830860776	0.0251	98.05	93.99
ValRS-m-RNAi -3	52672542	7953553842	52363114	7763866450	0.0252	98.03	93.91
Control-1	50124534	7568804634	49822112	7398407852	0.0251	98.05	93.98
Control-2	57088444	8620355044	56772964	8424814783	0.0249	98.12	94.17
Control-3	57892400	8741752400	57526316	8540745020	0.0249	98.11	94.15

Table S2. Primers used in this study

Transcript	Forward Primer (5′–3′)	Reverse Primer (5′–3′)
RP49	CGGTTACGGATCGAACAAGC	CTTGCGCTTCTTGGAGGAGA
ValRS-m	CTAATTCAGCGGCGCAACTC	CGGCTCCGTGATCTCTACAC
Aly	GACTCCTTCCTGGACAAGCC	GATGAAGGCGGCCGAAAATC
Cbc	GAAGGATCGCTACGGACCAG	TCGCTCGATGAGAATGGTGG
Tsen54	ACGCCCTCTGCTTTGAAGAT	AGTGGTGCGGTGAGAAGATG
Wuc	AGTACACTTTGGAGGAGGACC	GCGGGAGTAGACTTAGTTCAT
Comr	CTATGCAGGAATTGTGCGGC	TCTTCGCATTCGCTGGAACT
Topi	AGGAAGCCAGCATCCACTTC	GGTCCGTGTTGGCAAACTTC
Knon	ATAATACGCAGCGAGGAGGC	TGTCTCTTGCGCTCCAATGT
VhaM9.7-d	GAGGAGCGTGGTCTAATCCG	GTGTGGCTGGATTCAACTGC
Ocn	TAGGTTCGGCAGGACCATTG	ACCCTCCACCCAGGCATTTG
Tengl4	GCAGTAACGGGAGCA	GTTGACGGCATTGATAG
Poldip2	CGTGGCAGGAGAAGAACCAT	CAGTAGCGCCACCAGTAGAC
Vha100-3	ACGGAGCACTACTACCGACT	ACCAATCACTGTGCTCTCCG
CG31913	GATAAGGAGCAGGAGCCGAC	CCGTATGAACGTGAGGCACT
SdhAL	GAGGACAAAGCCACGGAGAA	ATGTTTGGTCATGGTCCGCT
Fig	CTGTTTGAGAAACTGGCCGC	CACCACCGTGACGAGATAGG
CG3092	GCGAGCCTGACTTCTTGG	CGTGTCGTTGCGGGATA
ND-B14.5AL	ATGTTTGGTCATGGTCCGCT	CCGAGGATCCCTGGTGTAGT
Sprn	GATCGAGCACCACGGATCAT	ATGTAGTCGATGCGCCACTT
mics1	ACCAGAGGTCCTTCCCTCAA	GTAGCACAGACCCACGAGAC
Dic3	TCCGTGGAAGTACCTGGTGA	GCACTCTGCTGACGGATGAT
tomboy40	GTACTTGCAGTCCGTCACCA	CAAAGTGCAAGACCACACGG
ttm3	CGGCCTGGTGTGGATTACTT	GTGGCACCTCTTACTAGCCG
GC2	TGGAGTGGCATGCGTCTATC	CCTTCGCTGGCGATAGTCTT
mAcon2	TGTGCCAGCATAGCCAATGA	TCTATAATGCCGTCCCGTGC

UQCR-11L	CAGGCCAAAGGTCACATTGC	TCAGCGACGAAGTCGAACAA
cyt-c-d	GTGATGCAGAGAACGGCAAG	TGTCCAGGTAACGCCCTTCT
mEFTu2	ACCTACGCTCACACGGATTG	TGGCAGCCACCACTAGAATG
cyt-c-1L	TTCTCCCTGCTCACCGGATA	TCCACTTGAGGAAGACGCAC
CysRS-m	TCAGTTGATGGTGACGGCTC	TCAGTTGGGCCTTTAACGCT
OXA1L	TGCACTGCCACCTAAGAAGG	CGAATCGGATCTCGTCCAGG
Gpo3	AACCGCACCCATTCTATT	AACCGCACCCATTCTATT
mEFTs	CCTTCAAGCGGTTCGTT	GATGGTGGCATTCTCCC
Mpc1	TAGCAAAGCGGTAGCA	ACCTTGTAGGCAAATCG
Mul1	AGTTGCGTAACCAGAGCC	TCCAGTTCACCAATAGCG
